# An audit addressing the quality of prescribing sodium valproate in early intervention service

**DOI:** 10.1192/bjo.2021.214

**Published:** 2021-06-18

**Authors:** Humaira Aziz, Khushboo Khatri, Sneha Upadhyaya

**Affiliations:** 1 Birmingham Community Healthcare NHS Foundation Trust; 2 University hospital Birmingham

## Abstract

**Aims:**

This Audit aims to review prescribing practice concerning Valproate in early intervention services.

**Method:**

The audit was undertaken across four EI hubs in Birmingham. Audit standards were derived from POMH-UK (Prescribing Observatory for Mental Health) QIP. Drug cards of the entire EIS caseload in November 2020 were reviewed to identify patients on any preparation of Valproate. A total of 31 patients were identified. Electronic notes of all the patients on Valproate were reviewed to compare prescribing practices against national standards.

**Result:**

A total of 31 patients were prescribed sodium Valproate. All these patients had target symptoms documented in their notes. Reason for starting Valproate was mostly documented as agitation and aggression rather than elation in the mood. In was unclear if patients had full physical health checked before starting Valprotae as in majority (94%) valproate was commenced as an inpatient. Not all cases had detailed inpatient discharge notes making it difficult to fully understand the rationale for starting Valproate.

55% of the patients were on an off-license valproate preparation. Where used off-license majority (93%)of these patients had no documentation of the rationale behind off-license use. Similarly, in most cases (93%)there was no evidence of off-license use being discussed with the patients. Most patients had received adequate monitoring in the community (74%) although there was limited documentation of screening for common side effects. Prescribers were noted to be using Semi-sodium Valproate and Sodium Valproate interchangeably despite these not being bioequivalent.

**Conclusion:**

We recommend that

1. Periodic treatment reviews should document the assessment of response and screening for side effects.
2. Where used clinician should clearly discuss and document the off-license use with patients. 500 mg Semi-sodium valproate (Depakote) is approximately equivalent to 433 mg Sodium Valproate (Epilim). If switching from Semi-sodium Valproate to Sodium Valproate, a slightly higher (approximately 10%) dose of Sodium Valproate should be used.
3. Unless clear evidence of affective illness is identified, the ongoing need for Valproate should be regularly reviewed by the clinicians.

